# Therapeutic Potential of Polyphenols-Loaded Polymeric Nanoparticles in Cardiovascular System

**DOI:** 10.3390/molecules25153322

**Published:** 2020-07-22

**Authors:** Olga Pechanova, Ezgi Dayar, Martina Cebova

**Affiliations:** Institute of Normal and Pathological Physiology, Centre of Experimental Medicine, Slovak Academy of Sciences, 81371 Bratislava, Slovakia; ezgi.dayar@savba.sk (E.D.); martina.cebova@savba.sk (M.C.)

**Keywords:** hypertension, atherosclerosis, heart failure, ROS, nitric oxide, polymeric nanoparticles, resveratrol, quercetin, curcumin, cherry extracts

## Abstract

Numerous studies document an increased production of reactive oxygen species (ROS) with a subsequent decrease in nitric oxide (NO) bioavailability in different cardiovascular diseases, including hypertension, atherosclerosis, and heart failure. Many natural polyphenols have been demonstrated to decrease ROS generation and/or to induce the endogenous antioxidant enzymatic defense system. Moreover, different polyphenolic compounds have the ability to increase the activity/expression of endothelial nitric oxide synthase (eNOS) with a subsequent enhancement of NO generation. However, as a result of low absorption and bioavailability of natural polyphenols, the beneficial effects of these substances are very limited. Recent progress in delivering polyphenols to the targeted tissues revealed new possibilities for the use of polymeric nanoparticles in increasing the efficiency and reducing the degradability of natural polyphenols. This review focuses on the effects of different natural polyphenolic substances, especially resveratrol, quercetin, curcumin, and cherry extracts, and their ability to bind to polymeric nanoparticles, and summarizes the effects of polyphenol-loaded nanoparticles, mainly in the cardiovascular system.

## 1. Introduction

Scientific research in recent years has placed significant focus on exploring the beneficial effects of natural polyphenols in the prevention and treatment of cardiovascular and neurodegenerative diseases and, in particular, cancer [1,2,3]. Polyphenols belong to a group of powerful antioxidants that supplement and enhance the function of endogenous antioxidants and enzymes involved in defensive action against the increased oxidative load caused by overproduction of reactive oxygen species (ROS) [3,4]. Moreover, many polyphenolic compounds have been shown to increase the activity/expression of endothelial nitric oxide synthase (eNOS) with subsequent enhanced nitric oxide generation [5,6].

Serious findings suggest that overproduction of ROS and oxidative stress accompany different cardiovascular diseases, including hypertension, atherosclerosis, and heart failure (for a review, see Reference [7]). Thus, reducing oxidative stress may protect and improve cardiovascular and metabolic functions through different cellular and molecular mechanisms. A number of articles assert that increased superoxide generation with subsequent decreased nitric oxide (NO) bioavailability is the most important cause of endothelial damage and impaired endothelium-dependent relaxation, which leads to hypertension, atherosclerosis, and heart failure [8,9].

## 2. ROS/NO Disbalance in Cardiovascular Diseases

### 2.1. Hypertension

Nicotinamide adenine dinucleotide phosphate (NADPH) oxidase, namely, Nox1, Nox2, Nox4, and Nox5 isoforms, have been identified as the main sources of ROS in vascular cells during hypertensive conditions [9,10,11]. Both pharmacological inhibition and genetic deletion of NADPH oxidase lead to lowering blood pressure in the animal models of hypertension [7,11,12]. Nox5 seems to play an important role in human health, and disease in particular [10]. In addition, further potential sources of enzymatic ROS production, e.g., xanthine oxidase, mitochondrial electron transport chain, lipoxygenase, cyclooxygenase, peroxidases, hem oxygenases, and uncoupled endothelial NO synthase (NOS), have been documented to significantly contribute to the oxidative stress in hypertension [7,9,13]. Superoxide-mediated oxidation of BH4, and deficiency of the substrates l-arginine and S-glutathionylation, have been described as the main molecular mechanisms of NOS uncoupling [14,15,16]. Similarly, decreased activity of antioxidant enzymes like superoxide dismutase (SOD), catalase, glutathione peroxidase, or glutathione reductase importantly contributes to oxidative stress during increased blood pressure [7,17].

ROS may also activate proinflammatory nuclear factor kappaB (NF-κB)-dependent pathways and increase levels of cytokines, such as interleukin 1 (IL-1) and tumor necrosis factor (TNF)-α, with subsequent phosphorylation of tyrosine kinases and inhibition of eNOS activity (for a review, see Reference [18]). On the other hand, activation/delivery of eNOS to different hypertensive modes may prevent or reduce increased blood pressure [9,19]. Moreover, NO has been shown to antagonize the vasoconstrictor and proliferative effects of angiotensin II (Ang II), while it decreases sodium excretion and the expression of the angiotensin-converting enzyme (ACE) and angiotensin AT1 receptor [20]. Recently, platelet-derived NO was demonstrated to play an important role in the regulation of platelet function and the adhesive process in hypertensive patients [21].

### 2.2. Atherosclerosis

The atherosclerotic process in the arterial wall is characterized by ROS-mediated oxidation of low-density lipoproteins (LDL) cholesterol to oxidized (ox) LDL cholesterol [22,23]. Products of lipid peroxidation, such as malondialdehyde, oxidized phospholipid, and 4-hydroxynonenal, belong to highly reactive species and lead to the generation of oxidation-specific epitopes (OSEs) [24]. ROS may potentiate OSE sensing by increased expression of endothelial TLR2, TLR4, and lectin-like oxidized LDL receptor-1 [24,25]. Damage to the arterial wall is characterized by excessive fibrosis of the intima and fatty plaque formation due to LDL/oxLDL cholesterol accumulation, proliferation of smooth muscle cells, and infiltration and/or migration of monocytes, T cells, and platelets. Penetration of the cells into the vascular wall is conditioned by the expression of leukocyte and chemokine adhesion molecules, in which the transcription is performed by NF-κB. Proinflammatory molecules, such as interleukin 6 (IL-6), interleukin 18 (IL-18), and TNF-α cytokines, adhesion molecules, matrix metalloproteinases (MMPs), and C-reactive protein (CRP) produced by monocytes, macrophages, and/or adipose tissue further potentiate microinflammation and the oxidative load [26,27].

Endothelial NO has the opposite role in this process, decreasing expression of the adhesion molecule and inhibiting endothelial–leukocyte interaction and cytokine-induced NF-κB activation [28,29]. Activation/targeted delivery of endothelial NOS and SOD to endothelial cells decreases leukocyte adherence, inhibits NF-κB activation, and reduces adhesion molecule expressions in different in vitro and in vivo experiments [30,31]. Similarly, platelet-derived NO may significantly contribute to the reduction of the adhesive process [21].

### 2.3. Heart Failure

Increased NADPH oxidase activity has also been demonstrated during conditions leading to heart failure. The most common causes of increased NADPH oxidase activity include enhanced production of Ang II, endothelin-1, or TNF-α, and increased mechanical stretch [32,33,34]. Nox2 and Nox4 have been identified as the main isoforms in cardiomyocytes in heart failure. Nox4, localized primarily within the mitochondria, seems to be mainly responsible for increased ROS generation [34]; however, diaphragms from patients with heart failure have shown Nox2 expression and p47phox phosphorylation highly associated with elevated protein oxidation [35]. Moreover, aldosterone-dependent activation of Nox2 significantly contributes to the profibrotic effect of Ang II in the heart [33]. Deficiency of NADPH oxidase has been shown to protect the heart from left ventricular remodeling and dysfunction after myocardial infarction [36]. Uncoupled eNOS has also been shown to further increase ROS production, leading to left ventricular remodeling, left ventricular dilatation, and contractile dysfunction [37].

In contrast, the functional dimer of eNOS with normal NO production may improve contractile function and decrease interstitial fibrosis in the impaired myocardium [38,39]. Indeed, organic nitrates, including nitroglycerin (GTN) and isosorbide mono (ISMN) and dinitrate (ISDN), have been used in cardiovascular medicine for 150 years. These drugs reliably ensure vasodilator activity necessary for the treatment of coronary artery disease and heart failure [39]. It was also demonstrated that nebivolol, belonging to the third generation of β-blockers with NO-dependent properties, and the NO donor LA-419 may improve left ventricular function and reduce left ventricular hypertrophy at doses that do not affect arterial blood pressure [9,39,40].

## 3. Effects of Natural Polyphenolic Substances on ROS/NO Disbalance

**Figure 1:**
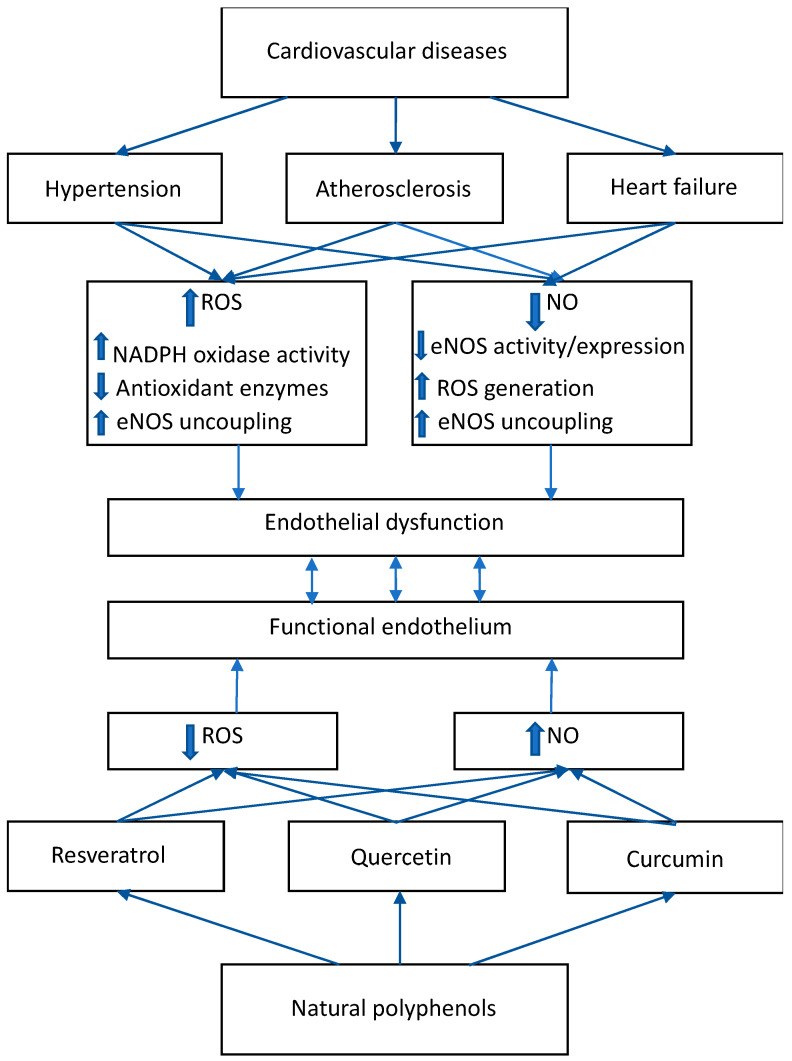
Cardiovascular diseases, including hypertension, atherosclerosis, and heart failure are accompanied by increased production of reactive oxygen species (ROS), also via enhanced nicotinamide adenine dinucleotide phosphate (NADPH) oxidase activity, with subsequent decreased nitric oxide (NO) generation, leading to endothelial dysfunction. Many natural polyphenols like resveratrol, quercetin, or curcumin are able to fight endothelial dysfunction by their ability to decrease ROS generation, induce the endogenous antioxidant enzymatic defense system, and increase activity/expression of endothelial NO synthase (eNOS).

Natural polyphenols generally have low bioavailability and different kinetic restrictions; thus, the direct free radical scavenger activity of these substances is very limited. There are a number of studies documenting the rather indirect antioxidant effect of polyphenols. Many polyphenols have been demonstrated to modify expression of different genes and to induce the endogenous antioxidant enzymatic defense system [41,42,43] (Figure 1). For example, treatment with chlorogenic acid, a dietary polyphenol, has been shown to decrease NADPH oxidase activity in spontaneously hypertensive rats (SHR) [44] and to prevent decreased activity of SOD, catalase, glutathione peroxidase, and glutathione-S-transferase in isoproterenol-induced myocardial oxidative stress in the rat myocardium [45]. Similarly, the beneficial cardioprotective effect of malvidin by restoring catalase, SOD, and glutathione peroxidase has been documented in a similar model of myocardial infarction [46]. On the other hand, kaempferol improved cardiac function via the activation of extracellular signal-regulated protein kinases 1 and 2 (ERK1/2) and the inhibition of p38 and *C*-jun *N*-terminal kinase (p38/JNK)/TNF-α/NF-kB/p65 pathways in the model of myocardial ischemia-reperfusion injury [47]. Another well-known polyphenol, quercetin, has the ability to attenuate postconditioning myocardial ischemia/reperfusion injury in rats through the activation of the phosphatidylinositol 3-kinase (PI3K)/Akt pathway [48].

Resveratrol has a variety of cardiovascular-beneficial effects. In SHR, it has been shown to decrease both NADPH oxidase activity and overexpression of Nox2, Nox4, and p47phox [49]. It has been identified as a direct and indirect sirtuin 1 (SIRT1) activator [50,51] with an increasing effect on SIRT1 expression as well [52]. Resveratrol has been shown to activate nuclear factor erythroid 2-related factor (Nrf2) indirectly [53] and upregulate miRNA-21, thus exerting cardioprotective effects in cardiac remodeling and apoptosis [54]. Moreover, it has been shown to activate eNOS by stimulating the membrane estrogen receptor [55]. Specifically, a subpopulation of estrogen receptor (ER) α activated by resveratrol is bound with caveolae in the endothelial membrane and coupled to the eNOS through a G protein [56]. The diarylheptanoid, curcumin, also has important vascular protective effects. Curcumin treatment significantly delayed the onset of stroke and increased the survival of stroke-prone spontaneously hypertensive rats independently of blood pressure reduction. Upregulation of the NO pathway has been identified as a mechanism responsible for this preventive process [57]. Moreover, curcumin reduces NF-κB, affecting gene regulation, and decreases the TNFα-induced expression of intercellular adhesion molecule-1 (ICAM-1), monocyte chemoattractant protein-1 (MCP-1), and IL-8 mRNA, characteristic of the atherosclerosis process [58]. Similarly, extracts from different cherries have been documented to be capable of favorable interactions with the risk factors of atherosclerosis. Besides their positive effects on the lipid spectrum and glycemia, reduction of ROS and improvement of endothelial dysfunction (Figure 1) have been demonstrated in both in vitro and in vivo experiments [59,60]. Recently, a new inverse molecular docking approach was used to identify potential human protein targets of curcumin and resveratrol, which could provide further insights into molecular mechanisms of antioxidant and anti-inflammatory actions of polyphenolic compounds [61,62].

Thus, the effects of polyphenolic compounds and their molecular actions in the cardiovascular system are different depending on both the type of polyphenol and respective cardiovascular disease. The absorption and bioavailability of natural polyphenols are, however, too low, and can be a serious obstacle for the beneficial actions of these substances. To remedy the low bioavailability, low solubility, and rapid degradability of polyphenols, different approaches have been developed with the aim to transport the respective polyphenolic substance throughout the gastrointestinal tract and deliver it to the targeted tissue. Recent progress in delivering polyphenols to the targeted tissues is characterized on three carrier levels, namely liposomes, microemulsion, and nanoparticles (for a review, see Reference [42]). Among them, biopolymeric nanoparticles represent a promising strategy for the protection and effective targeted delivery of natural polyphenolic substances.

## 4. Polymeric Nanoparticles

Recently, the use of polymeric nanoparticles has been based on nonbiodegradable polymers, such as polyacrylamide, polystyrene, and poly (methyl) methacrylate [63]. For such particles, inflammatory responses and chronic toxicity were observed, and therefore, research has focused on biodegradable polymeric nanoparticles with reduced toxicity, higher biocompatibility, and a better ability to regulate drug release kinetic patterns. With the exception of natural polymers like chitosan, albumin, alginate, and gelatine, the synthetic polymers mainly include poly (lactide) (PLA), poly (lactide-co-glycolide) copolymers (PLGA), poly (ε-caprolactone) (PCL), and poly (amino acids) [64,65,66,67]. These biodegradable synthetic polymers should fulfil two major requirements: performance and safety. They have to have the maximum biocompatibility to have a therapeutic effect, and biodegradation in a timeframe compatible with the healing of the target system or tissue. At the same time, they should be safe, which means not inducing in vivo toxicity and not promoting an inflammatory response in the immunological system [68].

Firstly, polyethylene glycol (PEG)-coated synthetic copolymers conjugated with active mediators have been shown to yield drug delivery systems with positive properties [69,70]. PEG coatings form a hydrated ring which prevents protein interactions and reduces opsonization, resulting in an increased circulation time and lower activation of the immune system [71]. Furthermore, a conjugation of glycoprotein Ib (GPIb) to PLGA nanoparticles has been shown to increase nanoparticle adhesion to the targeted surface, cellular uptake of nanoparticles, and controlled release of the active substances (for a review, see References [72,73]). PLA is relatively hydrophobic, which allows it to be used for implants like stents, screws for bone fixations, but also for drug delivery systems [74]. Polymeric nanoparticles have been reported to cross the intestinal barrier after oral administration and therefore, it is effectively used for oral drug delivery. It is generally accepted that the cells predominantly involved in the capture of nanoparticles administered intravenously are the macrophages of liver and spleen and circulating monocytes. The polymeric nanoparticles, along with toxins and unmetabolized nutrients, are passed through the kidney and taken up by the glomerular or peritubular capillaries, and eventually eliminated from the body by renal clearance [75].

Nowadays, targeted nanoparticle delivery systems in the field of cardiovascular disease are under intensive investigation. Minimizing the side effects while maximizing the drug effectiveness by targeted delivery poses a challenge not only in atherosclerosis, but also in hypertension, myocardial infarction, and heart failure (for a review, see References [72,73]). First, a liposome drug delivery system has been proven to be a successful option for the treatment of angina pectoris. Encapsulated amiodarone, an anti-arrhythmic drug, in conventional liposomes demonstrated a reduced morality rate due to arrhythmia and negative hemodynamic changes in rat models of cardiac ischemic/reperfusion procedure [76]. Later, treatment of aliskiren (a renin inhibitor)-loaded PLA nanoparticle decreased the blood pressure of SHR much more significantly than the powdered form [77]. Similarly, nanostructured lipid carriers and solid lipid nanoparticles improved the oral bioavailability of a calcium channel blocker, nisoldipine [78]. PLGA and PCL seem to be effective delivery systems for nifedipine and felodipine since they significantly reduced blood pressure in hypertensive rats [79,80]. Innovative NO-releasing polymeric nanomaterials are among the new potential solutions in the development of qualitatively new antihypertensive drugs [81]. Osako et al. demonstrated that the PEG–PLGA copolymer is able to deliver a NF-κB decoy oligodeoxynucleotide, which is directed against the NF-κB binding site in the promoter region [82]. This copolymer has been demonstrated to prevent monocrotaline-induced NF-κB activation in a rat model of monocrotaline-induced pulmonary arterial hypertension [83].

Still, the key problem with using nanoparticles is their toxicity. The small size and large surface area to volume ratio makes them very reactive. Nanoparticles may even generate ROS and other free radicals, resulting in an increased oxidative load and inflammation [84]. Recently, natural polyphenols-loaded nanoparticles have been the focus of interest thanks to their antioxidant properties, which additionally may exceed the prooxidant effects of some nanoparticles. Among them, resveratrol, quercetin, curcumin, and cherry extracts are the most frequently studied.

## 5. Therapeutic Effects of Polyphenols-Loaded Polymeric Nanoparticles

### 5.1. Effects of Resveratrol-Loaded Nanoparticles

Resveratrol (3,5,4′-trihydroxystilbene) belongs to non-flavonoid polyphenolic compounds, specifically, to the stilbenoid group [85,86]. Resveratrol has two structural isoforms—*cis* and *trans*. Trans-resveratrol is known as the most abundant and active form of resveratrol [86,87]. This, a natural plant-derived polyphenol, is contained mainly in red grapes and wine [87], berries, peanuts, and soy [88,89].

Preclinical studies have demonstrated that resveratrol has beneficial and protective effects on cardiovascular diseases, but also on diabetes, obesity, cancer, and neurodegenerative diseases. As a result of its pharmacological activities, resveratrol is classified as an antioxidant with anti-inflammatory, anti-carcinogenic, anti-aging, and cardioprotective properties [86,88]. Resveratrol has been shown to decrease the oxidative load by affecting antioxidant enzymes such as SOD, catalase, glutathione reductase, glutathione peroxidase, glutathione transferase, and oxidoreductases. Moreover, it increases eNOS production, inhibits lipid peroxidation [86,90], mitogen-activated protein (MAP) kinases and iNOS activities [91], and has anti-inflammatory effects through the downregulation of proinflammatory mediators like COX-1 and COX-2 [92]. Increased eNOS activity/expression is the main protective effect of resveratrol against vascular damage in cardiovascular diseases. Moreover, in endothelial cells, iNOS expression is regulated by NF-κB, which is inhibited by resveratrol [93]. Furthermore, resveratrol can also protect the cardiovascular system by modulating lipoprotein metabolism, inhibiting platelet aggregation [94,95], regulating vascular smooth muscle cell proliferation [91], inhibiting TNF-α [96], and upregulating tumor suppressor p 53 [86].

The limitations associated with the pharmacological use of this polyphenol are poor water solubility, the short biological half-life, chemical instability, and rapid metabolism [86,97]. Thus, nanoparticle delivery systems represent an ideal way to transport resveratrol to target tissues and ensure sufficient bioavailability [86,98]. Resveratrol-loaded polymeric nanoparticles are no bigger than 100 nm, so they can easily pass through the membrane and be internalized by cells. Resveratrol-loaded polymeric nanoparticles provide controlled drug release, increased drug solubility, drug targeting, and drug protection from degradation [86,89]. In the study by Singh and Pai [99], resveratrol-loaded PLGA nanoparticles had better oral bioavailability and absorptivity in rats in comparison with the pure drug [99]. Similarly, Siu et al. [100] documented that resveratrol-loaded galactosylated PLGA nanoparticles had better bioavailability and in vitro anti-inflammatory activity in rats and lipopolysaccharides-induced macrophage cell line RAW 264.7 cells, respectively [100]. Using carboxymethyl chitosan to prepare resveratrol-loaded nanoparticles provoked improvements in resveratrol antioxidant activity and bioavailability after oral administration in rats. Compared to the pure drug, resveratrol-loaded nanoparticles exhibited increased in vivo absorption, prolonged duration of action, and increased relative bioavailability by 3.5 times [101]. Oral administration of resveratrol loaded into N-trimethyl chitosan conjugated with palmitic acid nanoparticles in Balb/c mice provided a 3.8-fold increase in resveratrol bioavailability compared to the pure drug. This increase was attributed to the muco-adhesive and high absorption effects of the polymeric nanoparticles, as well as to the ability to prevent resveratrol degradation [89]. Cheng et al. [102] reported that dual-shell polymeric nanoparticles, multistage continuous targeted drug delivery carrier (MCTD)-NPs, which utilize a multistage continuous targeted strategy to deliver ROS scavengers specifically to the mitochondria of ischemic cardiomyocytes, increased the distribution of resveratrol in the ischemic myocardium and reduced infarct size in myocardial ischemia/reperfusion injury in rats [102]. Hardy et al. [103] used resveratrol-loaded multifunctional poly (glycidyl methacrylate) (PGMA) nanoparticles to study the effect in Langendorff I/R heart preparation. They reported that exposure of hearts to resveratrol-loaded nanoparticles was able to delay the release of creatine kinase and lactate dehydrogenase, the markers of an injured myocardium [103], (Table 1). Thus, resveratrol-loaded nanoparticles represent a promising tool for supportive treatment in cardiovascular diseases.

### 5.2. Effects of Quercetin-Loaded Nanoparticles

Quercetin (3,3′,4′,5,7-pentahydroxyflavone), a powerful antioxidant, belongs to the flavonoid family, and it is generally present as quercetin glycoside, especially in plants [104,105,106]. Quercetin is bound to mono- or oligo-saccharides through the glycosidic bonds with the 3′ hydroxyl group on the oxygen-containing ring [106]. Quercetin is widely contained in grapes, berries, apples, cherries, citrus fruit, red wine, black tea, onions, and tomatoes. Concentrations of quercetin may vary depending on the plant or even on the parts of the same plant [107]. Like other polyphenols, quercetin has limited water solubility. The bioavailability of the water-soluble derivative of quercetin is only 20% [108]. However, quercetin glycoside has a higher bioavailability than quercetin rhamnoside or quercetin galactoside [107].

Quercetin has important cardio-protective effects, including antioxidant, anti-inflammatory, anti-atherosclerotic, and anti-hypertensive effects. It also has preventive effects in dyslipidemia, endothelial dysfunction, and platelet aggregation [109]. Quercetin exerts its anti-inflammatory effects by inhibiting cyclooxygenase and lipoxygenase and decreasing prostaglandins and C-reactive proteins [110,111]. In diabetic rats, quercetin has been shown to improve dyslipidemia, decrease serum glucose levels, and reduce oxidative stress [112] by scavenging ROS and modulating antioxidant enzymes [113]. In spontaneously hypertensive rats, quercetin lowered blood pressure [114], reduced transcription of NF-κB [115], and decreased IL-6, IL-1ß, and TNF-α [116]. Dietary quercetin may modulate blood NO concentrations and inhibit NADPH oxidase activity [117].

The use of quercetin as a therapeutic target is, however, limited considering its poor aqueous solubility, instability in the physiological medium, and low bioavailability [107,118]. Therefore, polymeric nanoparticles are a good tool to increase bioavailability and reduce degradation of this flavonoid. Quercetin-loaded PLA nanoencapsulation demonstrated a higher water solubility and sustained release of the drug, leading to better bioavailability and stability of quercetin. On the other hand, the antioxidant capacities of the PLA-encapsulated quercetin and pure quercetin were almost the same [119]. Comparison of quercetin and catechin-loaded PLGA nanoparticles showed that quercetin was more slowly released from PLGA, probably due to the carbonyl and carboxyl interactions of the polymer and flavonoid molecules. Moreover, quercetin showed a higher radical scavenging activity compared to catechin [120]. Ghosh et al. [121] suggested that oral treatment with quercetin-loaded PLGA might play a protective role against oxidative damage in ischemia reperfusion induced in young and aged rats [121]. Wang et al. [122] layered a bioactive polymer (PLGA layers) onto superparamagnetic SiN to control the medication discharge profile. The PLGA layer on the outside of SiN can act as a gate-keeping layer to direct the medication discharge from SiN. They demonstrated that SiN@QC-PLGA nanobio-composite properties improve the practical similitude to the local myocardium, permitting cell enlistment, attachment, expansion, and articulation of heart proteins, which can be utilized in anticipation of atherosclerosis and other cardiovascular diseases [122] (Table 1). In addition, a novel system of polymeric PLGA nanoparticles loaded with quercetin and fabricated via the electrohydrodynamic atomization process may have great potential in the prevention of atherosclerosis and other relative cardiovascular diseases [118].

### 5.3. Effects of Curcumin-Loaded Nanoparticles

Curcumin (1,7-bis(4-hydroxy-3-methoxyphenyl)-1,6-heptadiene-3,5-dione), also called diferuloylmethane, is a polyphenolic compound contained in turmeric of the ginger family [123,124]. It is responsible for the intense yellow turmeric color. The rhizome of turmeric contains between 1.5% and 3% of curcumin [125]. The chemical structure of curcumin is composed of two aromatic ring systems including o-methoxy phenolic groups connected by α,β-unsaturated β-diketone moiety. Curcumin displays enolic and diketonic forms due to tautomerism between enol and keto structures [126,127].

Curcumin exhibits various beneficial physiological activities, including antioxidant, anti-inflammatory, and anti-proliferative effects. As a result of these therapeutic properties, curcumin has been shown to protect the heart against the development of cardiac hypertrophy, cardiotoxicity, and heart failure. It has beneficial effects in the atherosclerotic process and in diabetic cardiovascular complications [128]. Curcumin may improve oxidative stress, mitochondrial dysfunction, and inflammation through regulating various cell signaling pathways, including cytokines, chemokines, and growth factors and their receptors [123,129]. Curcumin exerts its anti-inflammatory effects via downregulation of NF-κB, resulting in decreased expression of TNF-α, IL-1, and IL6. Furthermore, curcumin inhibits the mitogen-activated protein kinase (MAPK) pathways [130] and activates lipoprotein lipase, peroxisome proliferator-activated receptor-gamma (PPAR-gamma), and PPAR-alpha [131,132]. The anti-proliferative effect of curcumin is supposed to be associated with its ability to induce heme-oxygenase-1 expression in vascular endothelial cells, vascular smooth muscle cells, and human aortic smooth muscle cells [133,134]. In atherosclerotic conditions, curcumin has been shown to reduce the serum levels of triglycerides, total cholesterol, LDL-cholesterol, and free fatty acids [135]. Curcumin can also prevent the activation of 3-hydroxy-3-methylglutaryl-coenzyme a reductase (HMG-CoA) via a transcriptional mechanism [136].

Like other natural polyphenolic compounds, clinical use of curcumin is limited because of its low bioavailability, poor absorption, and rapid metabolism [128]. Only 30–40% of the orally administered drug can be absorbed and most of the ingested curcumin is excreted in feces, unchanged. The absorbed curcumin is rapidly metabolized and only less than 0.02% of the metabolites are recovered from the liver, kidney, and body fat [137,138]. Several preclinical and clinical studies have proposed that the route of curcumin administration is an important factor in terms of its serum and tissue levels [138]. Curcumin-loaded polymer nanoparticle systems are therefore intensively studied [139,140].

The studies are focused on improving the solubility of the hydrophobic drug in the water, optimizing its stability, enhancing the pharmacokinetics, designing controlled release, preventing the drug from degradation, and targeting the organs. Using curcumin-loaded PLA–PEG copolymer nanoparticles, El-Naggar et al. [141] demonstrated that curcumin-loaded nanoparticles had better anti-inflammatory and antioxidant effects in a streptozotocin-induced diabetes model than pure curcumin [141]. In a similar model, curcumin-loaded chitosan nanoparticles promoted diabetic wound healing [142]. Carlson et al. [143] studied the cardio-protective effects of a combination of curcumin and resveratrol co-loaded into polymeric micellar in a cell model of doxorubicin-induced cardiotoxicity. The combination has been shown to markedly reduce apoptosis and ROS formation in the above cell model [143]. Similarly, curcumin-loaded copolymer PEG-Poly (ethylene glycol) methyl ether-block-poly (d, l lactide)-block-decane strongly inhibited apoptosis, lipid peroxidation, and production of NADPH-derived superoxides induced by exposure of cardiomyocytes to palmitate [144]. Curcumin-loaded to the same copolymer has been demonstrated to activate the AMP-activated protein kinase (AMPK)/mammalian target of the rapamycin complex-1/p-p70 ribosomal protein S6 kinase signaling pathway and regulate the expression of downstream proteins [145]. In a study by Nabofa et al. [146], the formulated curcumin-nisin-based PLA nanoparticles provided a significant level of cardio-protection in a guinea pig myocardial infarction model [146]. Curcumin encapsulated in carboxymethyl chitosan nanoparticles conjugated to a myocyte-specific homing peptide and successfully delivered to pathological myocardium was able to reduce cardiac hypertrophy and apoptosis in a rat model [147], and most importantly, in a double-blind randomized placebo-controlled clinical trial, nanocurcumin significantly decreased the levels of TNF-α, high-sensitivity C-reactive protein (hs-CRP), and IL-6 compared to placebo [148] (Table 1). Thus, using curcumin-loaded nanoparticles seems to have a prospective clinical future.

### 5.4. Effects of Cherry Extracts-Loaded Nanoparticles

Cherries are a rich source of polyphenolic compounds, especially anthocyanins, phenolic acids, and flavonols. Cyanidin-3-glucoside and cyanidin-3-rutinoside are the major anthocyanins in both sour and sweet cherries. The major phenolic acids in cherries are neochlorogenic acid, chlorogenic acid, and *p*-coumaric acid derivatives. The amount of flavonol quercetin-3-rutinoside is also significant. The concentrations of anthocyanins and phenolic compounds can vary depending on the cultivar, stage of ripening, storage conditions, and harvest time. Cherry fruit also contains several organic acids, including malic, citric, ascorbic, and fumaric acids [149,150,151]. It is a good source of carotenoids, potassium, tryptophan, serotonin, and melatonin [150].

Cherry fruit has been widely studied for its nutritional properties and beneficial effects. Because of the high content of anthocyanins, phenolic acids, and flavonols, cherries have antioxidant, anti-inflammatory, and vasodilatory properties. In vitro studies have demonstrated that natural polyphenol-rich sweet cherry extracts are able to protect endothelial cells from oxidative stress [152]. Cherry extracts and their bioactive components can improve cardiovascular performance. The potential cardioprotective effects of cherry extracts or isolated anthocyanins have been demonstrated by their ability to increase NO production and antioxidant status, reduce lipid oxidation, and inhibit inflammation [153,154,155]. Furthermore, the cornelian cherry that contains a high amount of polyphenols has anti-atherosclerotic properties mainly based on its ability to curtail the inflammation process and improve endothelial dysfunction [59]. Dayar et al. [156] documented that different types of cornelian cherries have better beneficial effects than the powerful antioxidant coenzyme Q10 in obese Zucker rats. In contrast to coenzyme Q10, cornelian cherries decreased cholesterol, LDL levels, and ROS production, while increasing eNOS activity/expression [156]. In a randomized controlled trial, Chai et al. [157] showed that tart cherries can lower the systolic blood pressure and LDL cholesterol levels in older adults. Moreover, plasma levels of C-reactive protein, malondialdehyde, and oxLDL levels were significantly decreased after 12 weeks of tart cherry consumption [157].

However, the low bioavailability of polyphenols contained in cherry extracts is the major problem in terms of their use for therapeutic purposes. They have a poor intestinal absorption because of oxidation in the intestinal tract and rapid metabolic degradation in the liver. Thus, studies focused on targeted transport have been at the center of research [158]. The encapsulation of cherry extracts in nanoparticles based on chitosan derivatives improved the intestinal absorption of cherry polyphenols and enhanced their antioxidant and anti-inflammatory activity in an in vitro model based on human umbilical vein endothelial cells (HUVECs) [152]. HUVECs have also been used to investigate the effects of Crognola (*Prunus avium* L.) cherry-extract-loaded nanoparticles based on two different chitosan derivatives. Cherry extract loaded into S-protected thiolated derivative has been shown to have a much more efficient protective effect on H_2_O_2_-induced oxidative stress and a reduction in ROS production. Generally, the nanoparticles with protected thiol groups have demonstrated higher protective effects [159]. On the other hand, PLGA-based cherry extract encapsulation showed similar antioxidant activity as compared with the free extract. PLGA nanoparticles, however, demonstrated a low cytotoxicity and could allow for the administration of higher cherry extract doses [160]. To maintain the quality of sweet cherries and improve their antioxidant properties, Ma et al. [161] proposed a new delivery and protective system by immersing sweet cherries in nitric oxide-releasing chitosan nanoparticles (GSNO-CS NPs). GSNO-CS NPs were able to reduce ROS and glutathione and increase the antioxidant enzyme activities and the levels of ascorbic acid [161]. It seems that cherry-extract-loaded nanoparticles represent a promising tool not only in clinical studies, but in the food industry as well. Although the data concerning the cherry extracts loading on nanoparticles are interesting and promising, all other polyphenols have been used as pure compounds, so the evaluation/comparison of loading capacity and efficacy between the different types of particles requires further studies and is currently not possible.

## 6. Conclusions

Different cardiovascular diseases, including hypertension, atherosclerosis, and heart failure, are accompanied by an increased production of ROS with subsequent decreased NO generation, leading to endothelial dysfunction. Many natural polyphenols are able to fight endothelial dysfunction through their ability to decrease ROS generation, induce the endogenous antioxidant enzymatic defense system, and increase activity/expression of eNOS. Different nanoparticles, including biodegradable polymeric nanoparticles, are able to increase the efficiency and reduce the degradability of natural polyphenols, thus increasing their beneficial abilities in the target tissues. Resveratrol, quercetin, curcumin, or cherry-extract-loaded polymeric nanoparticles have been shown to markedly reduce ROS formation, the inflammatory process, apoptosis, lipid peroxidation, cardiac hypertrophy, and even to delay myocardium injury due to ischemia/reperfusion. Nowadays, different copolymers and polymeric nanobio-composites are being developed with the aim of decreasing nanoparticle reactivity, toxicity, enhancing pharmacokinetics, and designing controlled release. They represent a promising tool for the delivery of natural polyphenols to target tissues and enhance their desirable effects, which is useful in the treatment of various diseases, including cardiovascular diseases.

## Figures and Tables

**Table 1:** An overview of polyphenol-loaded polymeric nanoparticles and their possible effects.

Polyphenol	Model of the Study	The Type of Polymeric Nanoparticle	The Effect of Polyphenol-Loaded Nanoparticle
Resveratrol	Male wistar rats	RSV-loaded PLGA NPs	Improve oral bioavailability of RSV [99]
	Sprague dawley rats Lipopolysaccharides-induced RAW 264.7 cells	RSV-loaded galactosylated PLGA NPs	Increase: Oral bioavailability Intestinal permeability Anti-inflammatory activity in RAW 264.7 cell model [100]
	Male sprague Dawley rats	RSV-loaded carboxymethyl chitosan NPs	Increase: Bioavailability and water solubility of RSV Antioxidant activity [101]
	Male balb/c mice	RSV-loaded *N*-trimethyl chitosan conjugated with palmitic acid NPs	Increase bioavailability Prevent RSV degradation [89]
	MI/RI injury rats	RSV-loaded dual-shell MCTD-NPs	Reduce infarct size [102]
	Male wistar rats Landerdorff I/R heart	RSV-loaded multifunctional poly (glycidyl methacrylate) (PGMA) NPs	Delay the release of the injured myocardium markers; creatine kinase and lactate dehydrogenase [103]
Quercetin	Different solutions	Que-loaded PLA NPs	Increase: Bioavailability Stability Water solubility Sustain release of Que [119]
	C3-BAS cell system	Que-loaded PLGA NPs	Decrease release of Que Increase radical scavenging activity [120]
	I/R induced rats	Que-loaded PLGA NPs	Increase protective role of oxidative damage [121]
	H9C2 cell	SiN@QC-PLGA	Increase biodegradation and water solubility of Que [122]
Curcumin	STZ-induced diabetes model	Cur-loaded PLA-PEG copolymer NPs	Increase: Anti-inflammatory effect Antioxidant effect [141]
	STZ-induced diabetes model	Cur-loaded chitosan NPs	Promote diabetic wound healing [142]
	Cell model of doxorubicin-induced cardiotoxicity	Cur and RSV co-loaded polymeric micellar	Reduce: Apoptosis ROS formation [143]
	H9C2 cardiomyocytes	Cur-PEG-PDLLA	Inhibit: Apoptosis Lipid peroxidation NADPH oxidases [144]
	H9C2 cardiomyocytes	Cur-PEG-PDLLA	Activate AMPK/mammalian target of rapamycin complex-1/p-p70 ribosomal protein S6 kinase signaling pathway [145]
	Myocardial infarction model of guinea pig	Cur-nisin based PLA NPs	Cardio-protection Decrease levels of H_2_O_2_, MDA, ROS [146]
	Cardiac hypertrophy rat model	Cur-loaded carboxymethyl chitosan NPs	Reduce: Cardiac hypertrophy Apoptosis [147]
	Double-blind randomized placebo-controlled clinical trial in obesity	Nano-curcumin	Decrease: TNF-α hs-CRP IL-6 [148]

Resveratrol: RSV, polymeric nanoparticles: NPs, myocardial ischemia-reperfusion injury rats: MI/RI rats, Quercetin: Que, poly (lactide): PLA, poly (lactide-co-glycolide) copolymers: PLGA, Ischemia-Reperfusion: I/R, Super magnetic nano-silica@Que-loaded PLGA: SiN@QC-PLGA, Streptozotocin: STZ, Curcumin; Cur, Polylactide-poly (ethylene glycol): PLA-PEG, Reactive oxygen species: ROS, Curcumin-loaded copolymer PEG-Poly (ethylene glycol) methyl ether-block-poly (d, l lactide)-block-decane: Cur-PEG-PDLLA, hydrogen peroxide: H_2_O_2_, malondialdehyde: MDA, tumor necrosis factor α: TNF-α, high sensitivity C-reactive protein: hs-CRP, interleukin-6: IL-6, human umbilical vein endothelial cells: HUVECs.
